# Air quality, meteorological variability and pediatric respiratory syncytial virus infections in Singapore

**DOI:** 10.1038/s41598-022-26184-0

**Published:** 2023-01-18

**Authors:** Meng Han Lee, Diyar Mailepessov, Khairunnisa Yahya, Liat Hui Loo, Matthias Maiwald, Joel Aik

**Affiliations:** 1 Environmental Epidemiology and Toxicology Division, National Environment Agency, 11 Biopolis Way #06-05/08, Helios Block, Singapore, 138667 Singapore; 2 Environmental Monitoring and Modelling Division, National Environment Agency, 40 Scotts Road, #13-00, Singapore, 228231 Singapore; 3 Department of Pathology and Laboratory Medicine, KK Women’s and Children’s Hospital, 100 Bukit Timah Road, Singapore, 229899 Singapore; 4 Duke-NUS Graduate Medical School, 8 College Road, Singapore, 169857 Singapore; 5 Department of Microbiology and Immunology, Yong Loo Lin School of Medicine, NUHS Tower Block, 1E Kent Ridge Road Level 11, Singapore, 119228 Singapore; 6 Duke-NUS Medical School, Pre-hospital and Emergency Research Centre, 8 College Road, Singapore, 169857 Singapore

**Keywords:** Environmental sciences, Diseases, Risk factors

## Abstract

Respiratory syncytial virus (RSV) is an important cause of respiratory illness among children. While studies have focused on the air-quality and climate dependence of RSV infections, few have been undertaken in South-East Asia where the burden of respiratory illness is among the highest across the globe. This study aimed to determine the relationships between climatic factors and air quality with RSV infections among children in Singapore. We obtained all laboratory-confirmed reports of RSV infections in children below 5 years old from the largest public hospital specializing in pediatric healthcare in Singapore. We assessed the independent cumulative effects of air quality and meteorological factors on RSV infection risk using the Distributed Lag Non-Linear Model (DLNM) framework in negative binomial models adjusted for long-term trend, seasonality and changes in the diagnostic systems. We included 15,715 laboratory-confirmed RSV reports from 2009 to 2019. Daily maximum temperature exhibited a complex, non-linear association with RSV infections. Absolute humidity (Relative Risk, 90th percentile [RR_90th percentile_]: 1.170, 95% CI: [1.102, 1.242]) was positively associated with RSV risk. Higher levels of particulate matter of aerodynamic diameter of less than (i) 2.5 µm (PM_2.5_), (ii) 10 µm (PM_10_), carbon monoxide (CO) and sulfur dioxide (SO_2_) were associated with lower RSV infection risk. RSV infections exhibited both annual and within-year seasonality. Our findings suggest that falls in ambient temperature and rises in absolute humidity exacerbated pediatric RSV infection risk while increases in air pollutant concentrations were associated with lowered infection risk. These meteorological factors, together with the predictable seasonality of RSV infections, can inform the timing of mitigation measures aimed at reducing transmission.

## Introduction

Respiratory syncytial virus (RSV) is an important cause of respiratory illness. It has been estimated to cause approximately 34 million new acute lower respiratory infections (ALRI) among children below 5 years old each year^1^, with more than 3.4 million requiring hospitalization, and between 66,000 and 199,000 cases resulting in death^2^. The health burden of lower respiratory infections is particularly high in South-East Asia where the incidence rate is estimated at 17.8 per 1000 children per year^3^.

Climate change is expected to bring about more intense and frequent extreme weather events such as warmer and/or fewer cold days and nights, heat waves and heavy precipitation events, as well as droughts with increased duration and/or intensity^4^. Previous studies have reported short-term associations between RSV infections and meteorological factors. Temperature has been reported to be negatively associated with RSV among young children in Tunisia, Malaysia, China as well as Rome and Bologna, Italy^5–8^. The number of rainy days has been reported to be positively associated with RSV in Malaysia^5^. Humidity has been reported to be positively associated with RSV in Rome, Italy, and Singapore^7,9^. In terms of seasonal patterns, Fodha et al.^6^ reported that RSV outbreaks occur periodically every winter in temperate climates. In Malaysia, a tropical country, infections with RSV occur as epidemics from November to January^5^.

Apart from meteorological influences, air quality has also been reported to influence the risk of RSV infections. Particulate matter of aerodynamic diameter of less than 2.5 µm (PM_2.5_), Particulate matter of aerodynamic diameter of less than 10 µm (PM_10_), nitrogen dioxide (NO_2_)_,_ sulfur dioxide (SO_2_) and carbon monoxide (CO) were reported to be positively associated with RSV infections in Rome and Bologna, Italy, as well as Hangzhou, China^7,8,10^. While several studies have focused on these air quality influences in temperate settings, few have been undertaken in the tropics where the climate differs. Understanding the interconnection among these factors and RSV infections would aid in the formulation and development of evidence-based measures aimed at reducing the disease burden.

We aimed to determine the relationships between meteorological factors and air quality with RSV infections among children in Singapore. We hypothesized that ambient temperature was negatively associated with childhood RSV infections while absolute humidity and air pollutants were positively associated with childhood RSV infections.

## Methods

### Ethics statement

This study was granted approval by the Environmental Health Institute of the National Environment Agency (NEA), Singapore. All methods were performed in accordance with the relevant guidelines and regulations (e.g., the Declaration of Helsinki). Informed consent was granted by the parent/guardian to collect nasopharyngeal swabs from the patient for clinical diagnosis; we used aggregated counts of confirmed infections in our study.

### Research setting

Singapore is a highly urbanized city-state located in Southeast-Asia. It experiences a tropical climate with high temperature and humidity, as well as abundant rainfall all year round. The largest referral center in Singapore dedicated to women’s and children’s healthcare is KK Women's and Children's Hospital (KKH), an 830-bed hospital specializing in obstetrics, gynecology, pediatrics and neonatology.

### Outcome measure

We used laboratory-confirmed RSV infections in children aged under 5 as our outcome measure. We obtained these from records from 2009 to 2019 from KKH, the largest public hospital specializing in healthcare for women and children in Singapore. This was before many respiratory infections drastically declined in 2020 due to COVID-19 pandemic response measures^11^. Duplicate positive samples from the same patients were excluded before analysis. Specimens were collected on flocked swabs, submitted in universal transport medium and tested within 12 h (h). Between 2009 and August 2012, the hospital used the Seeplex RV12 OneStep ACE multiplex PCR detection kit, then the Seeplex RV15 OneStep ACE detection kit (both Seegene Inc., South Korea). This was replaced in March 2018 with the Biofire Respiratory Panel 1.0 multiplex PCR, and in July 2019 with the Biofire Respiratory Panel 2.0 (both BioFire Diagnostics, Inc. Salt Lake City, Utah). Respiratory virus direct IF testing (D3 Double Duet DFA Respiratory Virus immunofluorescence kit, Diagnostic Hybrids, Athens, OH, USA) was performed during the entire period 2009–2019. Viral RNA was extracted following manufacturers’ instructions. Testing was based on physician requests. The Seeplex PCRs targeted 12 and 15 different respiratory viruses, the Biofire Respiratory Panel 1.0 and 2.0 targeted 17 and 18 different pathogens, respectively, and the Double Duet IF kit targeted 8 different viruses. All primer and probe sequences and IF antibodies were of a proprietary nature, supplied by the kit manufacturers.

### Environment data

Contemporaneous daily measures of meteorological and air quality variations representing the city-wide national averages across Singapore were obtained from the National Environment Agency. The meteorological data comprised daily maximum ambient temperature, minimum ambient temperature, mean ambient temperature, absolute humidity and total rainfall while the air quality data comprised ambient concentrations of PM_2.5_, PM_10_, ozone (O_3_), NO_2_, SO_2_ and CO.

### Statistical analysis

#### Core regression model

We use a negative binomial model to assess the short-term associations between the number of RSV infections and environmental conditions on a daily time scale. We established an initial core regression model by including linear, quadratic and cubic functions of time to control for the long-term trends. We modelled seasonal patterns using Fourier terms corresponding to annual, half-yearly, 4-monthly, 3-monthly, 2-monthly, monthly, fortnightly and weekly periodicities. We then used the Likelihood Ratio Test (LRT) to assess which pairs of Fourier terms to retain in the core model. We included categorical terms to control for the day-of-week and public holidays. We used a categorical variable to account for the potential change in RSV trends following the switches in diagnostic test methods.

#### Environmental factors

Studies have reported lagged environmental effects on RSV infections^8,10^. To model the delayed associations between meteorological and air quality variations with RSV infections, we used a distributed lag model to study the correlation between environmental changes and the incidence rate of RSV infections. Since the incubation period for RSV infections is between 2 to 8 days^12^, we included lagged terms for environmental factors up to 8 days.

We created cross-basis terms for each of the 11 environmental factors and fitted them with the core model in 11 separate models to evaluate the overall cumulative association of each of the environmental factors. We then included all significant environmental terms from each of the 11 models, while accounting for collinearity either by excluding some terms or by building separate models for the correlated terms, into a final regression model to obtain the fully adjusted effects of each environmental factor. We then used backward elimination to obtain the most parsimonious penultimate model.

Including highly correlated independent variables may lead to inaccurate effect estimates. Previous studies have reported high levels of correlation between air pollutants^13,14^. We determined the degree of correlation between environmental factors by computing Pearson correlation coefficients. An absolute value of 1.0 indicates perfect collinearity and a value of 0 indicates non-collinearity. We first assessed potential collinearity between air pollutants by computing Pearson correlation coefficients (Appendix, Table 1). Using a correlation value of 0.6 between factors as the maximum threshold, we then grouped environmental factors into initial models to minimize multi-collinearity. We then assessed collinearity among environmental factors by using variance inflation factor (VIF). A value of 1 indicated non-collinearity and a value exceeding 10 indicated serious collinearity^15^. Using a VIF of 2.5 for the variables as the maximum threshold^16^, we then checked for correlation among variables in the penultimate models which would warrant further investigation or correction.

The measure of effect for each environmental factor on RSV infections was expressed as a relative risk (RR). That is, the change in RSV infections associated with an increase in the corresponding independent factor referencing its median value. For non-linear associations, we computed the RR using either the 10th or 90th percentile values for the meteorological and air quality factors. Lastly, we added lags of the model deviance residuals corresponding to the observed serial correlation in partial autocorrelation function (PACF) plots in order to address any remaining autocorrelation before obtaining the final model with the fully adjusted effect estimates.

The final PM_2.5_ model, as well as PM_10_ and CO models which were fitted separately as they were correlated with PM_2.5_, is shown in Eqs. (1–3):

1 $$\begin{gathered} \log {\text{~}}E\left( {Y_{t} } \right) = {\text{~~~}}\beta _{0} + \beta _{1} t + \beta _{2} t^{2} + \beta _{3} t^{3} + \mathop \sum \limits_{{k = 1}}^{3} \left\{ {{\text{~}}\beta _{{4k}} \sin \left( {2\pi kt/365.25} \right) + \beta _{{5k}} \cos \left( {2\pi kt/365.25} \right)} \right\} \hfill \\ \quad \quad \quad \quad + \beta _{{6d}} dow_{{d = 1{\text{~}}to{\text{~}}6}} + \beta _{7} holidays + \beta _{{8{\text{s}}}} pcr_{{s = 1{\text{~}}to{\text{~}}3}} + MaxT_{{cb}} + AH_{{cb}} + \left( {PM_{{2.5}} } \right)_{{cb}} \hfill \\ \quad \quad \quad \quad + \left( {SO_{2} } \right)_{{cb}} + \mathop \sum \limits_{{j = 1}}^{J} \beta _{{8{\text{j}}}} Deviance{\text{~}}Residual{\text{~}}Lag_{j} + \log \left( {N_{t} } \right) \hfill \\ \end{gathered}$$

2 $$\begin{gathered} \log {\text{~}}E\left( {Y_{t} } \right) = {\text{~~~}}\beta _{0} + \beta _{1} t + \beta _{2} t^{2} + \beta _{3} t^{3} + \mathop \sum \limits_{{k = 1}}^{3} \left\{ {{\text{~}}\beta _{{4k}} \sin \left( {2\pi kt/365.25} \right) + \beta _{{5k}} \cos \left( {2\pi kt/365.25} \right)} \right\} \hfill \\ \quad \quad \quad \quad + \beta _{{6d}} dow_{{d = 1{\text{~}}to{\text{~}}6}} + \beta _{7} holidays + \beta _{{8{\text{s}}}} pcr_{{s = 1{\text{~}}to{\text{~}}3}} + MaxT_{{cb}} + AH_{{cb}} \hfill \\ \quad \quad \quad \quad + \left( {PM_{{10}} } \right)_{{cb}} + \left( {SO_{2} } \right)_{{cb}} + \mathop \sum \limits_{{j = 1}}^{J} \beta _{{8{\text{j}}}} Deviance{\text{~}}Residual{\text{~}}Lag_{j} + \log \left( {N_{t} } \right) \hfill \\ \end{gathered}$$

3 $$\mathrm{log }E\left({Y}_{t}\right)= {\beta }_{0}+{\beta }_{1}t+{\beta }_{2}{t}^{2}+{\beta }_{3}{t}^{3}+{\sum_{k=1}^{3}\left\{{ \beta }_{4k}\mathrm{sin}\left(2\pi kt/365.25\right)+{\beta }_{5k}\mathrm{cos}\left(2\pi kt/365.25\right)\right\}+{\beta }_{6d}{dow}_{d=1 to 6}+{\beta }_{7}holidays+{\beta }_{8\mathrm{s}}{pcr}_{s=1 to 3}+{MaxT}_{cb}+{AH}_{cb}+\left(CO\right)}_{cb}+{({SO}_{2})}_{cb}+\sum_{j=1}^{J}{\beta }_{8\mathrm{j}}{Deviance Residual Lag}_{j}+\mathrm{log}\left({N}_{t}\right)$$

Where $E\left({Y}_{t}\right)$ is the expected number of reported RSV infections at day t. ${\beta }_{0}$ is the intercept. $t$, ${t}^{2}$ and ${t}^{3}$ are linear, quadratic and cubic functions of time, respectively ($t$ takes on values of 1–4017). $k$ is the number of full seasonal cycles within the year and takes on values of 1–3. The effects of day-of-week, public holidays and multiplex PCR system on the outcome measure are represented by $dow$, $holidays$ and $pcr$ respectively. The lagged and non-linear effects of maximum temperature, absolute humidity and air quality were modelled through the cross-basis functions ${MaxT}_{cb}$, ${AH}_{cb}$, ${\left({PM}_{2.5}\right)}_{cb}$, ${\left({PM}_{10}\right)}_{cb}$, ${\left(CO\right)}_{cb}$ and ${({SO}_{2})}_{cb}$ with a maximum lag of 8 days and natural cubic splines of 3 df—the use of 3 df for the natural cubic splines have been reported in several studies^17–20^. $\mathrm{Log}\left({N}_{t}\right)$ is an offset term representing the logged daily child population estimates interpolated from the annual mid-year population census data obtained from the Department of Statistics, Singapore^21^.

We conducted sensitivity analysis by increasing the flexibility from 3 to 4 df and 5 df for each of the significant weather and air pollutant variables. We assessed statistical significance at the 5% level. All statistical analyses were performed using R software Version 3.6.3.

## Results

### Descriptive statistics

**Figure 1:**
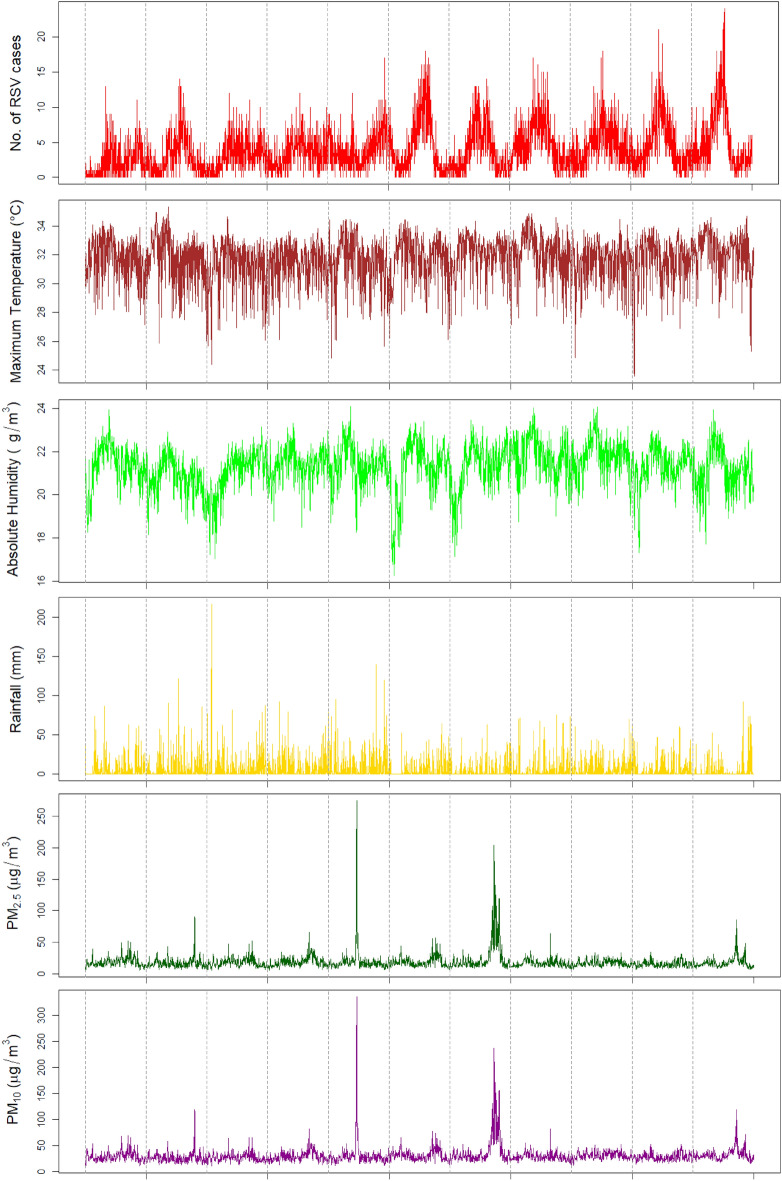

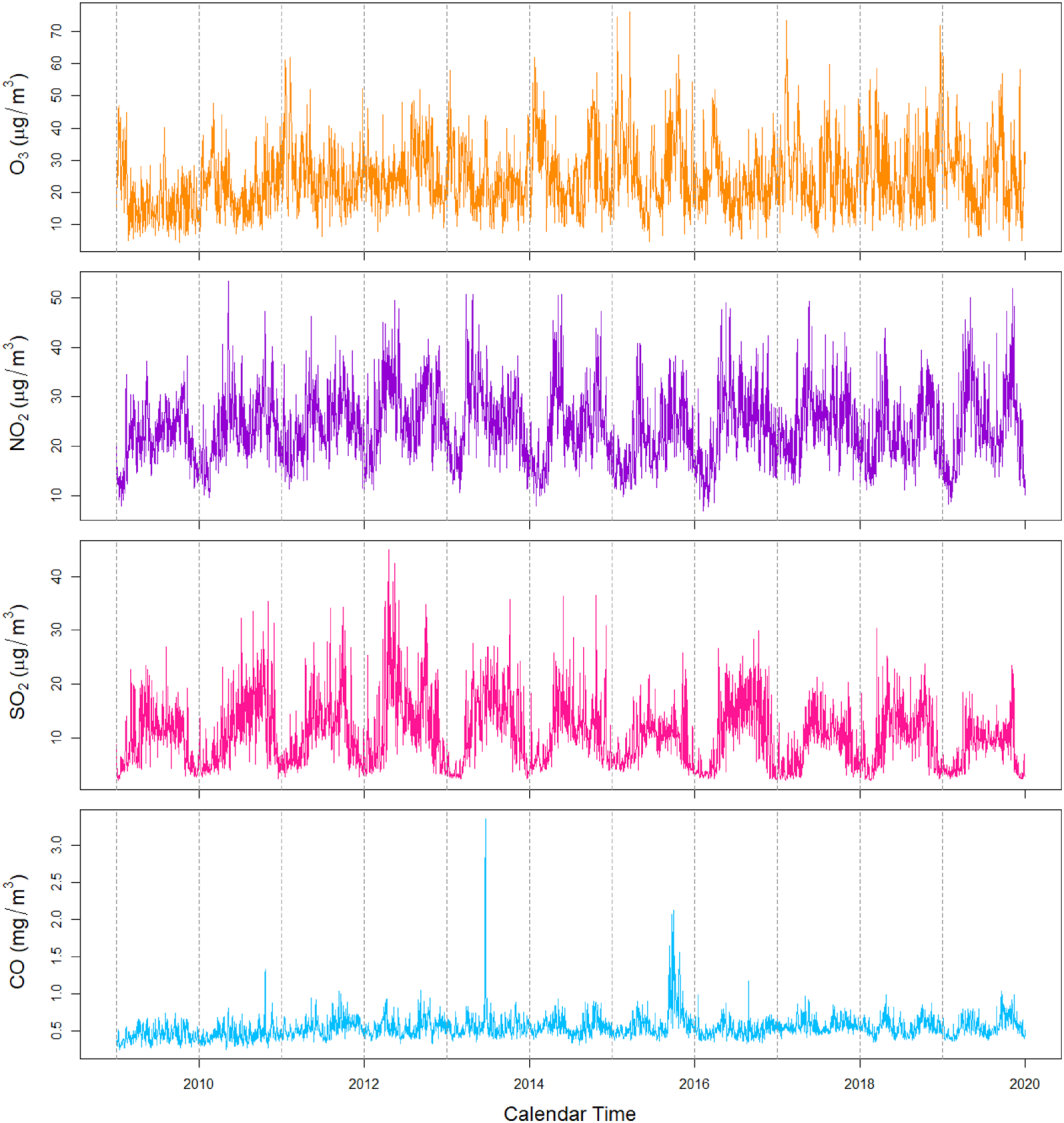
Dily measures of RSV incidence, meteorological factors and air quality from 2009 to 2019.

A total of 15,715 RSV infection cases were included in our study. The mean daily number of RSV infections was 3.9 (SD 3.2) (Table 1). There were 9065 (57.7%) RSV infections in males. Among all RSV reports, 2156 (13.7%) were confirmed by RT-PCR and 13,559 (86.3%) were confirmed by IF testing. The mean age of children with RSV infections was 1.2 years (SD: 1.0 year). Twenty-three (23) repeat positive results from the same patients were excluded. The mean daily maximum ambient temperature was 31.8 °C. The mean daily absolute humidity was 21.3 g/m^3^. Maximum ambient temperature peaked from March to May while absolute humidity peaked from April to June (Fig. 1). Cyclical variation was observed in the trend of SO_2_. PM_2.5_, PM_10_ and CO concentrations levels were the highest in October 2010, June 2013 and September–October 2015.

**Table 1:** Summary statistics of daily climatic factors, air pollution and RSV reports, 2009–2019.

Variables	Mean	SD	Min	Percentile			Max
				10th	50th (IQR)	90th	
RSV Reports	3.9	3.2	0	1	3 (2–6)	8	24
**Climatic factors**							
Max Temperature (^o^C)	31.8	1.5	23.6	29.7	32.0 (31.0–32.8)	33.4	35.3
Mean Temperature (^o^C)	27.9	1.1	23	26.4	27.9 (27.1–28.7)	29.3	31
Min Temperature (^o^C)	25.0	1.1	21.5	23.6	25.0 (24.3–25.8)	26.6	28.2
Absolute Humidity (g/m^3^)	21.3	1.0	16.2	20.0	21.4 (20.8–22.0)	22.5	24.1
Total Rainfall (mm)	5.3	12.5	0	0	0 (0–4)	18	216
**Air quality**							
PM_2.5_ (μg/m^3^)	18.3	12.6	5.1	10.6	15.8 (12.6–20.4)	26.2	274.4
PM_10_ (μg/m^3^)	30.2	15.5	9.7	19.5	27.5 (23.0–33.6)	40.4	335.9
O_3_ (μg/m^3^)	24.4	10.1	4.4	12.9	22.6 (17.2–30.2)	38.4	76.0
NO_2_ (μg/m^3^)	23.9	7.2	6.8	15.0	23.3 (18.8–28.5)	33.3	53.4
SO_2_ (μg/m^3^)	10.5	6.0	2.0	3.6	9.8 (5.4–14.0)	18.7	45.0
CO (mg/m^3^)	0.5	0.2	0.2	0.4	0.5 (0.4–0.6)	0.7	3.3

*SD* Standard deviation.

### Regression analysis

#### Long-term trend, seasonality and changes in diagnostic systems

**Figure 2:**
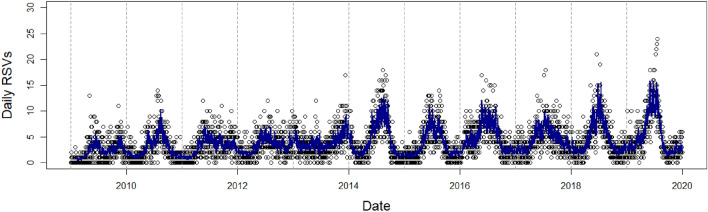
Time series of reported and predicted number of RSV infections, 2009–2019. The hollow circles represent the reported daily counts of RSV infections and the blue solid line represents the daily number of RSV infections predicted from the final PM_2.5_ model.

We observed a long-term increase in the daily number of RSV infections from 2009 to 2019 (*p* < 0.001) (Table 2). We found evidence of cyclical variations in the pattern of RSV infections (Fig. 2) at the 4-, 6- and 12-monthly intervals, with the highest peaks falling between June and August (Table 2). Each public holiday was associated with a 13.6% (RR: 0.864, 95% CI: 0.777–0.960) reduction in the risk of RSV infections. Changes in the diagnostic system were associated with differences in the number of reported infections (Appendix, Tables 5 and 6).

**Table 2:** Adjusted associations between public holidays and day-of-week with RSV infections in Singapore, 2009–2019.

Variable	β coefficient	RR	95% CI	*p* value (Wald test)	LRT *p* value
**Seasonal variation of RSV infections**					
*12−monthly*					< 0.001
Sine function	−0.24	–	−0.29 to −0.20	< 0.001	
Cosine function	−0.62	–	−0.68 to −0.56	< 0.001	
*6−monthly*					< 0.001
Sine function	−0.03	–	−0.07 to 0.01	0.163	
Cosine function	0.07	–	0.04 to 0.11	< 0.001	
*4−monthly*					< 0.001
Sine function	0.05	–	0.02 to 0.08	0.001	
Cosine function	0.04	–	0.01 to 0.06	0.006	
**Long−term trend of RSV infections**					
Linear function	0.00*	1.00*	1.00 to 1.00	< 0.001	
Quadratic function	0.00*	1.00*	1.00 to 1.00	< 0.001	
Cubic function	0.00*	1.00*	1.00 to 1.00	< 0.001	
**Day−of−week**					
Monday	Referent				< 0.001
Tuesday	−0.09	0.92	0.86 to 0.97	0.004	
Wednesday	−0.15	0.86	0.81 to 0.92	< 0.001	
Thursday	−0.22	0.80	0.75 to 0.86	< 0.001	
Friday	−0.23	0.79	0.74 to 0.84	< 0.001	
Saturday	−0.41	0.66	0.62 to 0.71	< 0.001	
Sunday	−0.36	0.70	0.65 to 0.74	< 0.001	
**Public holiday**					
No	Referent				
Yes	−0.15	0.86	0.78 to 0.96	0.007	
**Change in Multiplex PCR**					
Seegene RV12		Referent			< 0.001
Seegene RV15	0.23	1.26	1.15 to 1.38	< 0.001	
Biofire RP1.0	0.24	1.28	1.12 to 1.46	< 0.001	
Biofire RP2.0	−0.20	0.82	0.68 to 1.00	0.046	

*For the long-term trend of RSV infections, beta coefficients are above 0.00 for the linear and cubic terms, and below 0.00 for the quadratic term. Point estimates are above 1.00 for the linear and cubic terms, and below 1.00 for the quadratic term. *LRT* Likelihood Ratio Test.

#### Meteorological effects

**Figure 3:**
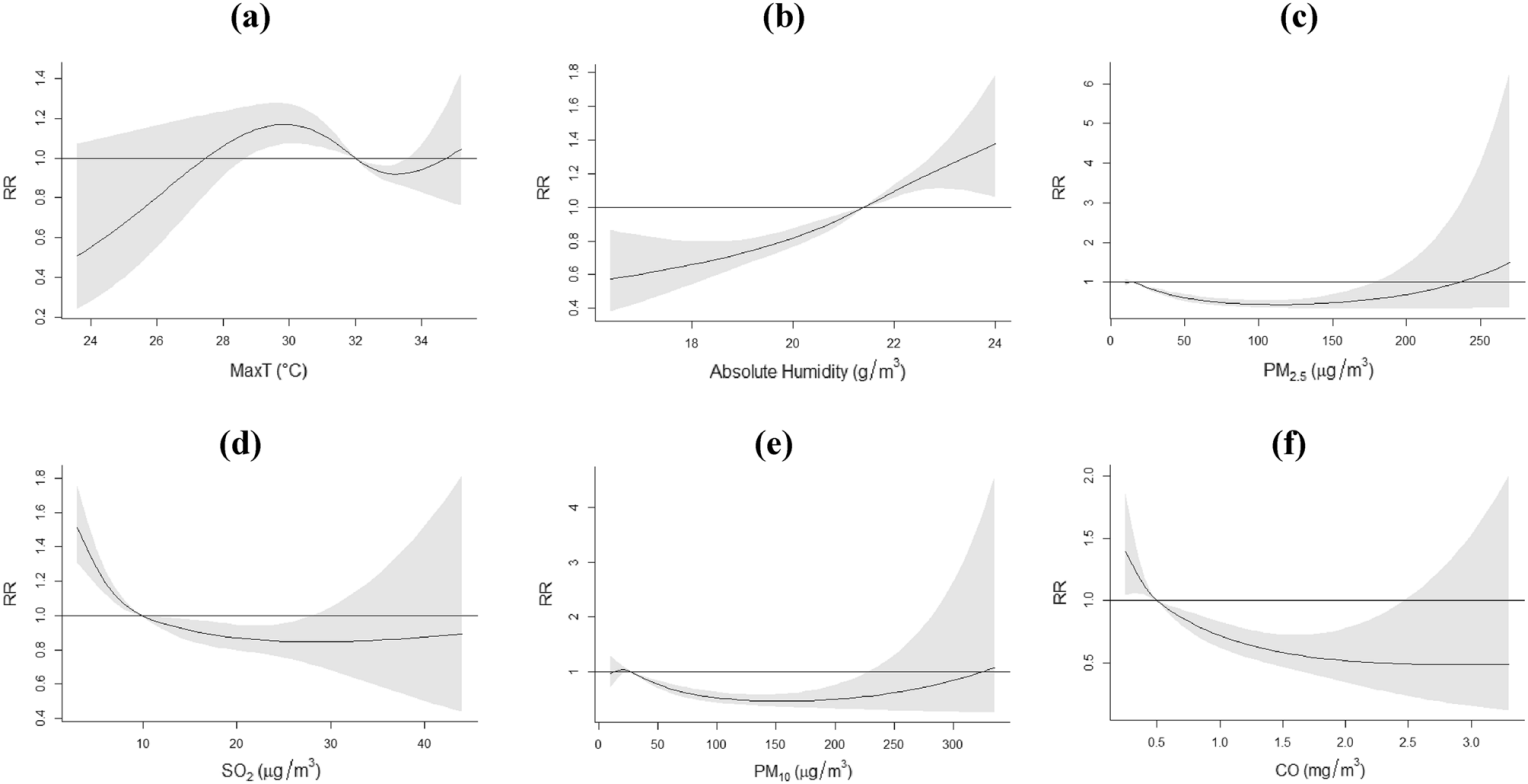
The cumulative effect of (**a**) maximum temperature (**b**) absolute humidity (**c**) PM_2.5_ (**d**) SO_2_ (**e**) PM_10_ and (f) CO on RSV infections over 8 days. (**a**–**d**) are from the final PM2.5 model, while (**e**) and (**f**) are from the PM_10_ model and CO model, respectively. Solid lines represent relative risk (RR), grey shaded areas represent 95% confidence intervals (CIs).

We observed a complex, non-linear cubic relationship between maximum temperature and RSV infections (Fig. 3a), though the association was non-significant towards the ends of the temperature range. From 29.7 °C (10th percentile) to 33.4 °C (90th percentile), which contains the maxima and the minima of the dose-response function, we observed a negative association between maximum temperature and RSV infection risk (RR_10th percentile_: 1.168, 95% CI: 1.068–1.277; RR_90th percentile_: 0.920, 95% CI: 0.863–0.981). We did not find any significant associations between RSV infections and minimum or mean temperature. Absolute humidity exhibited a positive, almost linear relationship with RSV infections (RR_90th percentile_: 1.170, 95% CI: 1.102–1.242) (Fig. 3b). Although we observed wider CIs when we increased the degrees of freedom for each of the weather variables, the shapes of the dose-response functions remained largely similar (Appendix, Fig. 7).

#### Air quality effects

Particulate matter was negatively associated with the risk of RSV infections. Ambient PM_2.5_ at the concentration of 26.2 μg/m^3^ was associated with a 16.3% cumulative decrease (RR_90th percentile_: 0.837, 95% CI: 0.794–0.884) in the risk of RSV infections (Fig. 3c) while ambient PM_10_ at the concentration of 40.4 μg/m^3^ was associated with a 14.8% cumulative decrease (RR_90th percentile_: 0.852, 95% CI: 0.807–0.900) in the risk of RSV infections (Fig. 3e). The risk of RSV infections declined with increases in CO concentration (RR_90th percentile_: 0.863, 95% CI: 0.801–0.929) (Fig. 3f). SO_2_ rises were associated with a 12.2% cumulative decrease (RR_90th percentile_: 0.878, 95% CI: 0.807–0.956) in the risk of RSV infections (Fig. 3d). We did not find any significant effect of nitrogen dioxide or ozone and thus did not retain either of them in our final models. Increasing the degrees of freedom for each of the air pollutant variables did not change the direction of the effects to a large extent, although we observed wider CIs (Appendix, Fig. 7). The pollutant and weather lag-specific effects may be found in the Appendix.

## Discussion

Few studies analyzing the influence of air pollution and climate variability on RSV infections in the tropics have been undertaken. In this study, we analyzed the relationship between meteorological factors and air quality and reports of RSV infections in Singapore. We found that increases in RSV infection risk were independently associated with declines in maximum temperature and increases in absolute humidity, while increases in air pollutant concentrations were associated with lower RSV risk.

### Meteorological effects

The negative association between daily maximum temperature and RSV infections in our study is biologically plausible and consistent with previous studies conducted in Hangzhou, China, and Maranhão, Brazil^8,22^. Lower temperatures enhance virus viability in the environment, thus leading to an increased risk of infection^5,8^. The lower heat makes the RSV lipid envelop more stable in the secretions through which it is transmitted, leading to increased virus activity at lower temperatures^7,23^. Lower temperatures may also enhance virus susceptibility by triggering changes in human physiology.

In our study, absolute humidity was positively associated with RSV infections. This finding is consistent with a previous study conducted in Hong Kong which found a positive and lagged association between vapor pressure (a proxy for absolute humidity) and RSV-related hospitalizations^24^. Absolute humidity was also positively associated with RSV infections in a study conducted in Mexico City and Miami^23^. Another study conducted in Rome also showed a higher number of RSV lower respiratory tract infection cases at higher relative humidity^7^. One possible mechanism through which higher humidity facilitates RSV infections is by increasing virus survivability. RSV is suspected to be transmitted via the aerosols and earlier studies showed higher RSV stability in large particles aerosols at higher humidity levels^7,9,23^.

### Particulate matter effects

In previous studies conducted in Hangzhou, China, and Poland, it was found that PM_2.5_ was positively associated with RSV infections^8,25^. PM_10_ was also positively associated with RSV infections in a study conducted in Bologna^10^. Higher PM levels can aid infections with RSV by impairing lung functions. These include increased bronchial hyperresponsiveness as well as weakened antimicrobial defense system of the lungs with increased exposure to PM_2.5_ and/or PM_10_^10,26^. Aside from the production of free radicals mentioned earlier, exposure to PM also decreases lung mRNA levels of antioxidant enzymes^27^. This further worsens the oxidative stress induced in the body, resulting in higher vulnerability to RSV infections. We hypothesized that particulate matter was positively associated with RSV infection risk. However, we observed a different direction of effect for particulate matter in our study. One plausible explanation for the inverse relationship observed in our study could be that parents (and consequently their children) might have chosen to increase their dwelling times indoors in their homes when PM levels were elevated. The National Environment Agency (Singapore) advises vulnerable persons, including children, to minimize strenuous outdoor activities when PM_2.5_ concentrations exceed 55 μg/m^3^ or when the Pollutant Standards Index value exceeds 100^28^. Spending less time in outdoor settings may have reduced their risk to RSV infections posed by other segments of the population outdoors. Another contributing reason for the negative association between RSV and PM_2.5_ may have been the effect of temperature. Average maximum temperature at high PM_2.5_ concentrations (≥ 90th percentile) was 0.6 °C lower than that at lower PM_2.5_ concentrations (< 90th percentile), suggesting that temperature could have exerted a negative effect on RSV infections. More studies are required to better understand the mechanism through which PM_2.5_ influences RSV infections.

### Carbon monoxide and sulphur dioxide exposures

In our study, CO was negatively associated with RSV infections. This was unexpected as other studies have found positive associations between CO and RSV infections in Hangzhou and Seoul^8,29^. However, a study by Tian et al.^30^ reported negative and lagged associations between CO and hospitalizations for respiratory tract infections. The antimicrobial and anti-inflammatory properties of CO, which may allow it to resolve the inflammation quickly after viral clearance, could explain its protective effects against RSV infections found in our study^30^. Experiments have also shown increased susceptibility to polymicrobial infections in heme oxygenase-1–deficient mice, likely due to lower endogenous CO production^31^. This suggests the possible benefit of CO in the inhibition of RSV infections. In addition, CO trends tended to follow those of particulate matter. It may be possible that CO might have been a proxy air pollutant species for the effects of particulate matter.

We observed a negative association between SO_2_ and RSV infections in our analysis. This was unexpected, considering that other studies have shown positive associations between SO_2_ and RSV infections in Seoul, Hangzhou and Paris^8,29,32^. One plausible explanation for the inverse relationship found in our study could be the virucidal property of SO_2_. An ambient SO_2_ concentration of 0.4 ppm was shown to inactivate aerosolized Venezuelan equine encephalomyelitis virus^33^. It is possible that the pollutant has similar virucidal effects on RSV. More studies are required to understand the biological mechanisms through which CO and SO_2_ exposure influence RSV infection risk.

### Seasonal pattern of RSV infections

The seasonal peak of RSV risk in Singapore occurred between June and August, coinciding with the Southwest Monsoon season. Other studies have reported different seasonal peaks in RSV infections, for instance in February in Tunisia and Italy, as well as in the months corresponding to the rainy season in Maranhão, Brazil^10,22^. It is well established in the literature that RSV epidemics vary between climates. In the tropics, infections peak in the rainy season, whereas in temperate climates, seasonal peaks occur in winters^6^. It is suggested that temperature and humidity play a bigger role in influencing the seasonal peaks in temperate and tropical regions, respectively^34,35^. The increased virus transmissibility as a result of indoor crowding due to the cold or rain could also account for peaks in winters and the rainy season^36^. In our study, the periodic middle of year RSV infection peaks did not coincide with the rainy season in Singapore but occurred after periodic rises in absolute humidity in the April/May period. We hypothesize that the periodic rise in absolute humidity may have led to a build-up of the virus in the human population, thus allowing transmission to be sustained for a period because of human-to-human transmission rather than meteorological factors.

### Public health implications

The relationships between RSV infections and environmental exposure that we observed in the tropical setting have important public health implications. The implementation of public awareness and personal hygiene initiatives before and during expected periods of relatively colder or more humid weather, particularly in the months of June to August, can be considered by public health authorities seeking to mitigate RSV transmission. Ventilation and setting indoor temperature and humidity to optimal levels may also help to reduce the probability of RSV transmission.

### Study strengths and limitations

There was a low probability of outcome misclassification in this study as we used laboratory-confirmed RSV infections. Nasopharyngeal swab testing was based on ordering by attending clinicians and is likely to reflect symptomatic infections rather than mild or asymptomatic cases. This may have contributed to some selection bias. The use of the distributed delay non-linear model enabled a more accurate characterization of the delayed and non-linear effects of air quality and meteorological exposures on RSV infections. We also conducted sensitivity analysis and found that the direction of effects remained similar regardless of modelling assumptions. We used data from a single major public hospital specializing in pediatric healthcare (estimated to account for 50% of all pediatric hospitalizations). Finally, as this was an ecological study, the results may not be generalizable to individuals. The use of the monitoring station data for the air pollution as a proxy of individual exposures was a limitation as well.

## Conclusions

Our findings suggest that falls in ambient maximum temperature and rises in absolute humidity increased pediatric RSV infection risk, while increases in air pollutant levels lowered the infection risk. These meteorological factors, together with the predictable seasonality of RSV infections, can inform the timing of mitigation measures aimed at reducing transmission. More studies are required to understand the mechanisms behind the air quality-RSV relationship.

## Supplementary Information

Supplementary Information.

## Data Availability

The RSV, air quality and weather data underlying the results presented in our study are owned by a third party. The data that support the findings of this study are available from KK Women's and Children's Hospital and the National Environment Agency, but restrictions apply to the availability of these data, which were used under license for the current study, and so are not publicly available. Data are however available from the authors (Contact_NEA@nea.gov.sg) upon reasonable request and with permission of KK Women's and Children's Hospital and the National Environment Agency.
