# Supplementary material for: Air quality, meteorological variability and pediatric respiratory syncytial virus infections in Singapore
Source: Sci Rep. 2023 Jan 18;13:1001. doi: 10.1038/s41598-022-26184-0 (PMC9848044; doi:10.1038/s41598-022-26184-0)
Supplement: Supplementary file 1 — Supplementary Information. [file 41598_2022_26184_MOESM1_ESM.docx]

**Air quality, meteorological variability and pediatric respiratory syncytial virus infections in Singapore**

Meng Han Lee^a+^, Diyar Mailepessov^a+^, Khairunnisa Yahya^b^, Liat Hui Loo^c^, Matthias Maiwald^c,d,e^ and Joel Aik^a,f^

**Appendix**

**Table 1**: **Pearson correlation coefficients for environmental factors**

|  | MaxT | AH | PM_2.5_ | PM_10_ | O_3_ | NO_2_ | SO_2_ | CO |
| --- | --- | --- | --- | --- | --- | --- | --- | --- |
| MaxT | 1 | 0.13 | 0.15 | 0.17 | 0.25 | 0.05 | 0.07 | 0.03 |
| AH |  | 1 | -0.01 | -0.05 | -0.38 | 0.38 | 0.18 | 0.17 |
| PM_2.5_ |  |  | 1 | 0.98 | 0.30 | 0.30 | 0.20 | 0.77 |
| PM_10_ |  |  |  | 1 | 0.36 | 0.26 | 0.14 | 0.76 |
| O_3_ |  |  |  |  | 1 | -0.18 | -0.16 | 0.07 |
| NO_2_ |  |  |  |  |  | 1 | 0.56 | 0.48 |
| SO_2_ |  |  |  |  |  |  | 1 | 0.14 |
| CO |  |  |  |  |  |  |  | 1 |

**Table 2**: **Generalized variance inflation factors for factors in final PM_2.5_ model.**

| **Covariate** | **GVIF** | **df** | **GVIF^(1/2*Df)** |
| --- | --- | --- | --- |
| MaxT | 4.28 | 15 | 1.05 |
| AH | 8.45 | 15 | 1.07 |
| PM_2.5_ | 6.26 | 15 | 1.06 |
| SO_2_ | 22.63 | 15 | 1.11 |

**Table 3: Generalized variance inflation factors for factors in final PM_10_ model.**

| **Covariate** | **GVIF** | **df** | **GVIF^(1/2*Df)** |
| --- | --- | --- | --- |
| MaxT | 4.65 | 15 | 1.05 |
| AH | 8.30 | 15 | 1.07 |
| PM_10_ | 5.43 | 15 | 1.06 |
| SO_2_ | 19.74 | 15 | 1.10 |

**Table 4: Generalized variance inflation factors for factors in final CO model.**

| **Covariate** | **GVIF** | **df** | **GVIF^(1/2*Df)** |
| --- | --- | --- | --- |
| MaxT | 3.78 | 15 | 1.05 |
| AH | 9.03 | 15 | 1.08 |
| CO | 5.81 | 15 | 1.07 |
| SO_2_ | 18.01 | 15 | 1.10 |

**Note: GVIF – generalized variance inflation factor, df – degrees of freedom.**

**Table 5: Adjusted associations between public holidays and day-of-week with RSV infections in Singapore from PM­_10­_ model, 2009 to 2019**

| **Variable** | **β coefficient** | **RR** | **95% CI** | **p-value  (Wald test)** | **LRT  p-value** |
| --- | --- | --- | --- | --- | --- |
| **Seasonal variation of RSV infections** |  |  |  |  |  |
| *12-monthly* |  |  |  |  | <0.001 |
| Sine function | -0.24 | – | -0.29 to -0.19 | <0.001 |  |
| Cosine function | -0.62 | – | -0.68 to -0.56 | <0.001 |  |
| *6-monthly* |  |  |  |  | <0.001 |
| Sine function | -0.03 | – | -0.06 to 0.01 | 0.206 |  |
| Cosine function | 0.07 | – | 0.04 to 0.10 | <0.001 |  |
| *4-monthly* |  |  |  |  | <0.001 |
| Sine function | 0.04 | – | 0.02 to 0.07 | 0.002 |  |
| Cosine function | 0.04 | – | 0.01 to 0.06 | 0.008 |  |
| **Long-term trend of RSV infections** |  |  |  |  |  |
| Linear function | 0.00* | 1.00* | 1.00 to 1.00 | <0.001 |  |
| Quadratic function | 0.00* | 1.00* | 1.00 to 1.00 | <0.001 |  |
| Cubic function | 0.00* | 1.00* | 1.00 to 1.00 | <0.001 |  |
| **Day-of-week** |  |  |  |  |  |
| Monday | Referent |  |  |  |  |
| Tuesday | -0.09 | 0.92 | 0.86 to 0.97 | 0.005 |  |
| Wednesday | -0.15 | 0.86 | 0.81 to 0.92 | <0.001 |  |
| Thursday | -0.22 | 0.80 | 0.75 to 0.86 | <0.001 |  |
| Friday | -0.23 | 0.79 | 0.74 to 0.84 | <0.001 |  |
| Saturday | -0.41 | 0.66 | 0.62 to 0.71 | <0.001 |  |
| **Public holiday** |  |  |  |  |  |
| No | Referent |  |  |  |  |
| Yes | -0.15 | 0.86 | 0.78 to 0.96 | 0.007 |  |
| **Change in Multiplex PCR** |  |  |  |  |  |
| Seegene RV12 | Referent |  |  |  |  |
| Seegene RV15 | 0.24 | 1.27 | 1.15 to 1.39 | <0.001 |  |
| Biofire RP1.0 | 0.24 | 1.27 | 1.11 to 1.45 | <0.001 |  |
| Biofire RP2.0 | -0.18 | 0.83 | 0.69 to 1.01 | 0.061 |  |

*For the long-term trend of RSV infections, beta coefficients are above 0.00 for the linear and cubic terms, and below 0.00 for the quadratic term. Point estimates are above 1.00 for the linear and cubic terms, and below 1.00 for the quadratic term. LRT: Likelihood Ratio Test

**Table 6: Adjusted associations between public holidays and day-of-week with RSV infections in Singapore from CO model, 2009 to 2019**

| **Variable** | **β coefficient** | **RR** | **95% CI** | **p-value  (Wald test)** | **LRT  p-value** |
| --- | --- | --- | --- | --- | --- |
| **Seasonal variation of RSV infections** |  |  |  |  |  |
| *12-monthly* |  |  |  |  | <0.001 |
| Sine function | -0.21 | – | -0.26 to -0.17 | <0.001 |  |
| Cosine function | -0.60 | – | -0.66 to -0.54 | <0.001 |  |
| *6-monthly* |  |  |  |  | <0.001 |
| Sine function | -0.06 | – | -0.10 to -0.01 | 0.009 |  |
| Cosine function | 0.08 | – | 0.04 to 0.11 | <0.001 |  |
| *4-monthly* |  |  |  |  | <0.001 |
| Sine function | 0.03 | – | 0.00 to 0.06 | 0.027 |  |
| Cosine function | 0.04 | – | 0.01 to 0.07 | 0.002 |  |
| **Long-term trend of RSV infections** |  |  |  |  |  |
| Linear function | 0.00* | 1.00* | 1.00 to 1.00 | <0.001 |  |
| Quadratic function | 0.00* | 1.00* | 1.00 to 1.00 | <0.001 |  |
| Cubic function | 0.00* | 1.00* | 1.00 to 1.00 | <0.001 |  |
| **Day-of-week** |  |  |  |  |  |
| Monday | Referent |  |  |  |  |
| Tuesday | -0.08 | 0.92 | 0.87 to 0.98 | 0.007 |  |
| Wednesday | -0.14 | 0.87 | 0.82 to 0.93 | <0.001 |  |
| Thursday | -0.21 | 0.81 | 0.76 to 0.86 | <0.001 |  |
| Friday | -0.22 | 0.80 | 0.75 to 0.85 | <0.001 |  |
| Saturday | -0.40 | 0.67 | 0.63 to 0.72 | <0.001 |  |
| Sunday | -0.36 | 0.70 | 0.66 to 0.75 | <0.001 |  |
| **Public holiday** |  |  |  |  |  |
| No | Referent |  |  |  |  |
| Yes | -0.15 | 0.86 | 0.77 to 0.96 | 0.005 |  |
| **Change in Multiplex PCR** |  |  |  |  |  |
| Seegene RV12 | Referent |  |  |  |  |
| Seegene RV15 | 0.23 | 1.26 | 1.14 to 1.38 | <0.001 |  |
| Biofire RP1.0 | 0.21 | 1.23 | 1.08 to 1.41 | 0.002 |  |
| Biofire RP2.0 | -0.28 | 0.76 | 0.62 to 0.92 | 0.006 |  |

*For the long-term trend of RSV infections, beta coefficients are above 0.00 for the linear and cubic terms, and below 0.00 for the quadratic term. Point estimates are above 1.00 for the linear and cubic terms, and below 1.00 for the quadratic term. LRT: Likelihood Ratio Test.


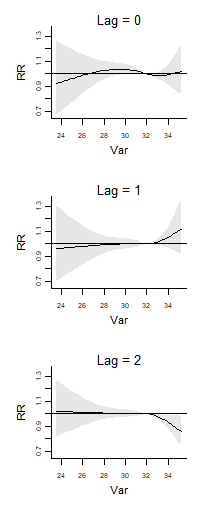

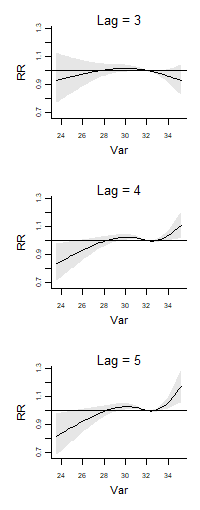

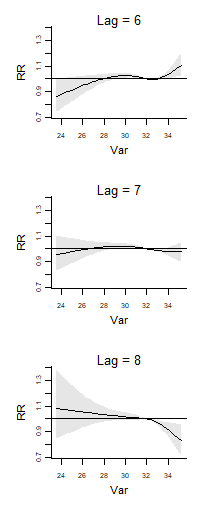
**Figure 1.** The lagged effect of maximum temperature on RSV from the final PM_2.5_ model. The RRs are derived from comparing each value on x-axis against its median. Shaded grey area denotes 95% CI.


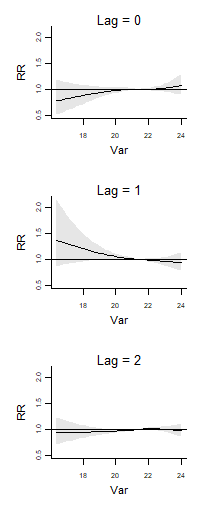

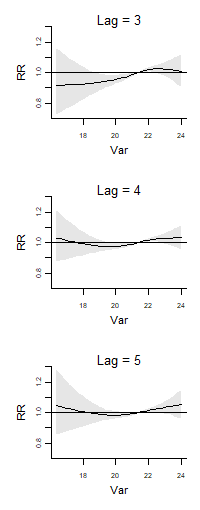

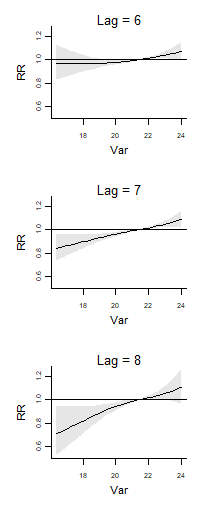
 **Figure 2.** The lagged effect of absolute humidity on RSV from the final PM_2.5_ model. The RRs are derived from comparing each value on x-axis against its median. Shaded grey area denotes 95% CI.


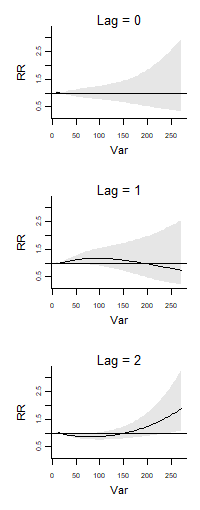

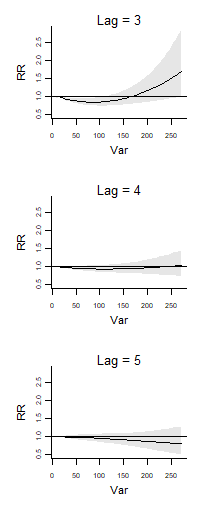

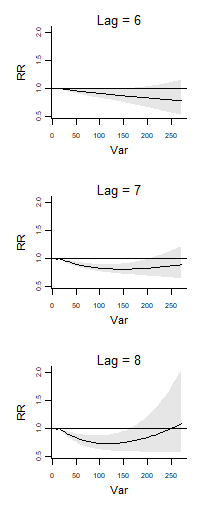
 **Figure 3.** The lagged effect of PM_2.5_ on RSV from the final PM_2.5_ model. The RRs are derived from comparing each value on x-axis against its median. Shaded grey area denotes 95% CI.


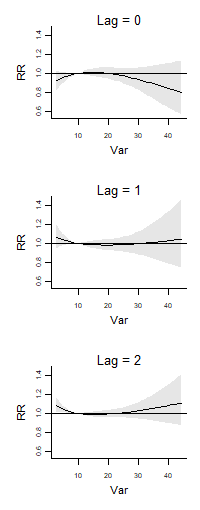

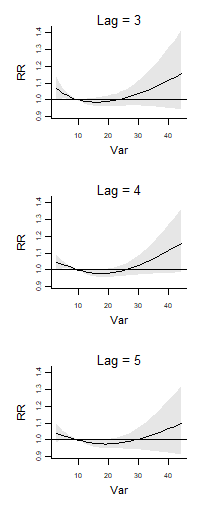

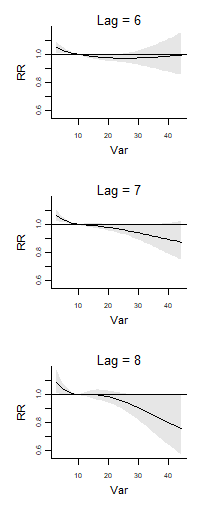
 **Figure 4.** The lagged effect of SO_2_ on RSV from the final PM_2.5_ model. The RRs are derived from comparing each value on x-axis against its median. Shaded grey area denotes 95% CI.


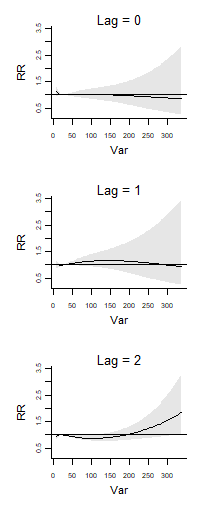

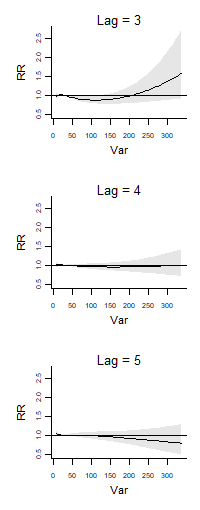

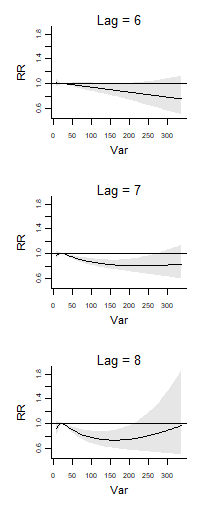
 **Figure 5.** The lagged effect of PM_10_ on RSV from the final PM_10_ model. The RRs are derived from comparing each value on x-axis against its median. Shaded grey area denotes 95% CI.


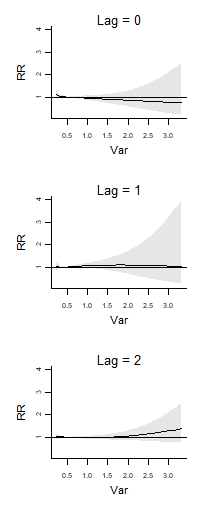

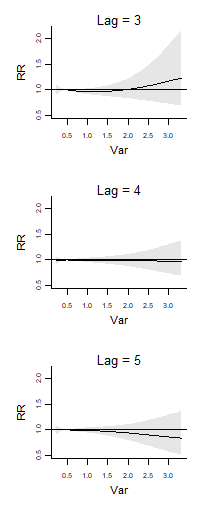

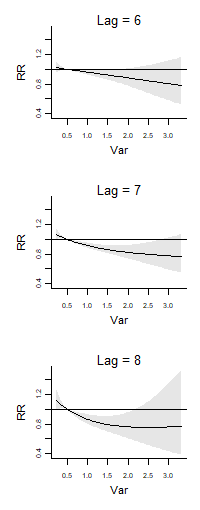
 **Figure 6.** The lagged effect of CO on RSV from the final CO model. The RRs are derived from comparing each value on x-axis against its median. Shaded grey area denotes 95% CI.

| **4 *df*** | **5 *df*** |
| --- | --- |
| 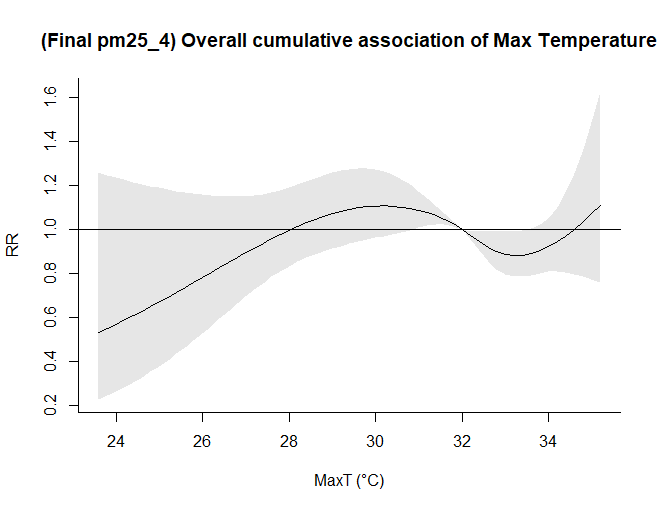 | 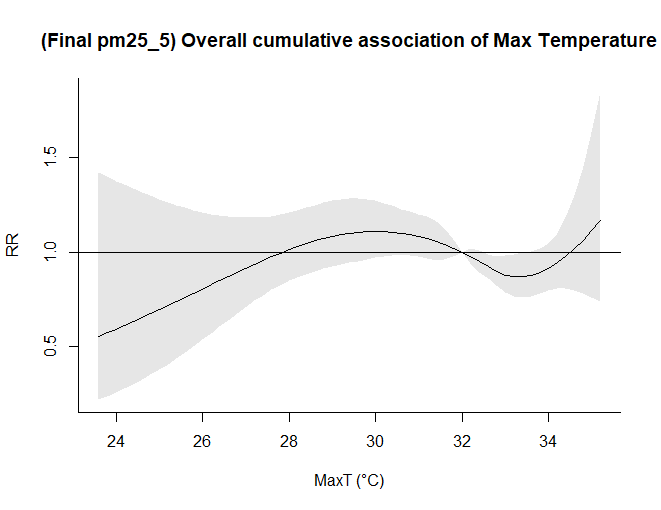 |
| [A] | [B] |
| 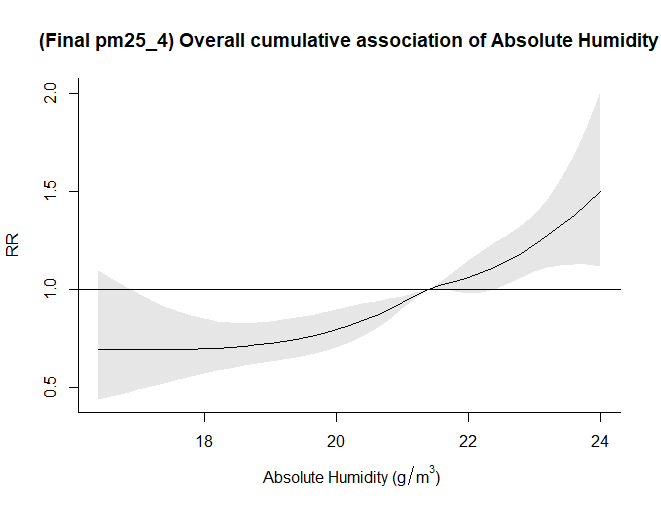 | 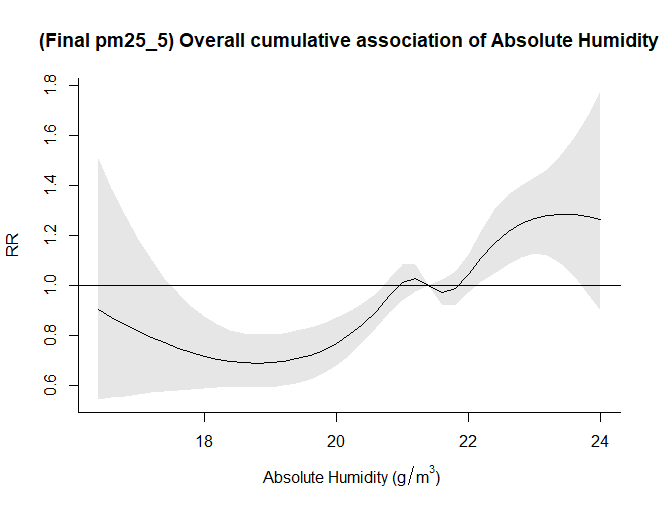 |
| [C] | [D] |
| 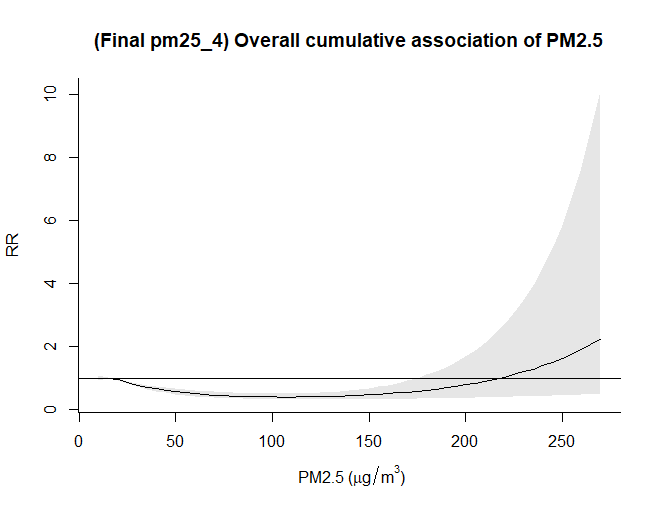 | 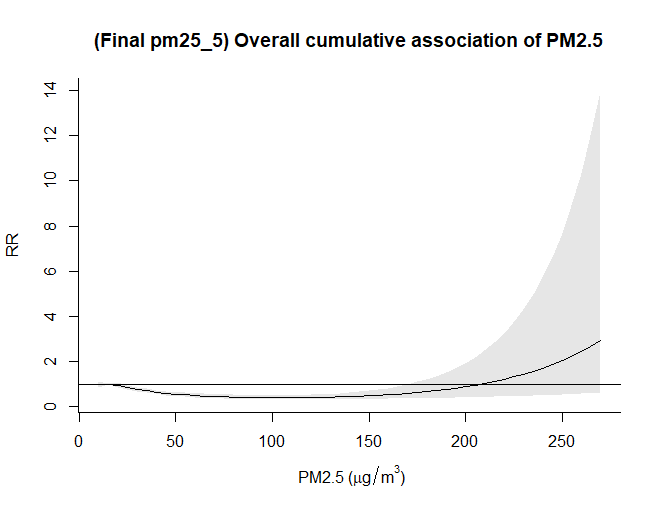 |
| [E] | [F] |
| 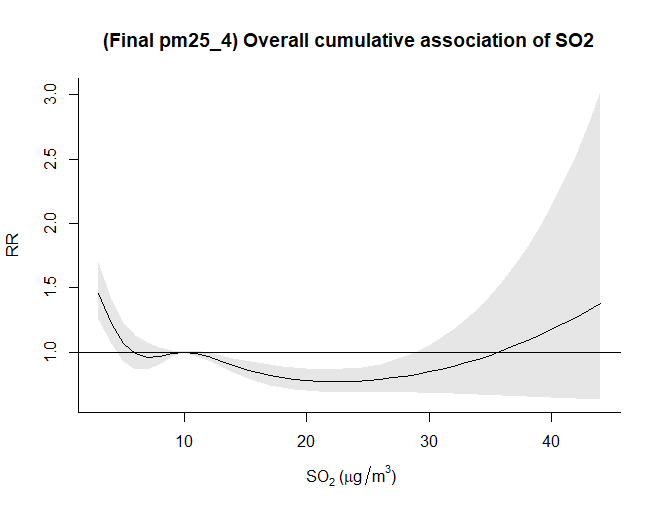 | 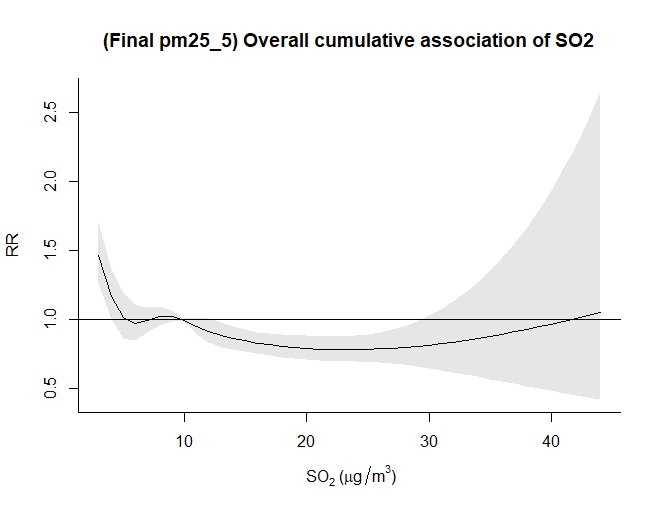 |
| [G] | [H] |

**Figure 7.** Sensitivity analysis using 4 and 5 degrees of freedom (*df)* for maximum temperature (A, B), absolute humidity (C, D), PM2.5 (E, F) and SO_2_ (G, H). Solid lines represent relative risk (RR), grey shaded areas represent 95% confidence intervals (CIs).

| **Before Adjusting for Autocorrelation** | **After Adjusting for Autocorrelation** |
| --- | --- |
| 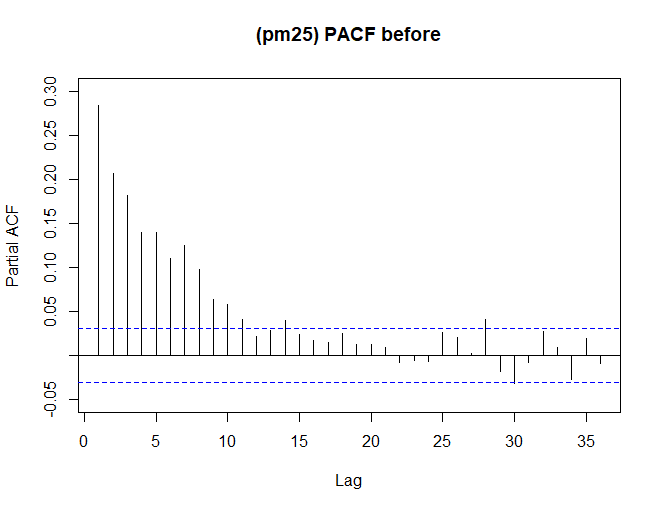 | 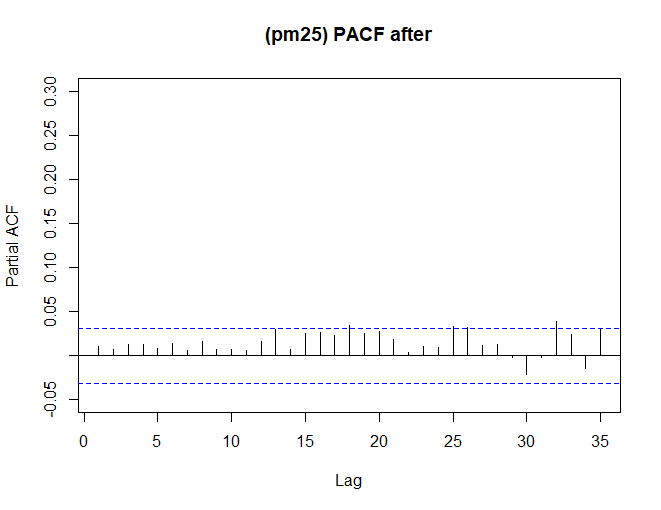 |
| [A] AIC: 17501 | [B] AIC: 16412 |
| 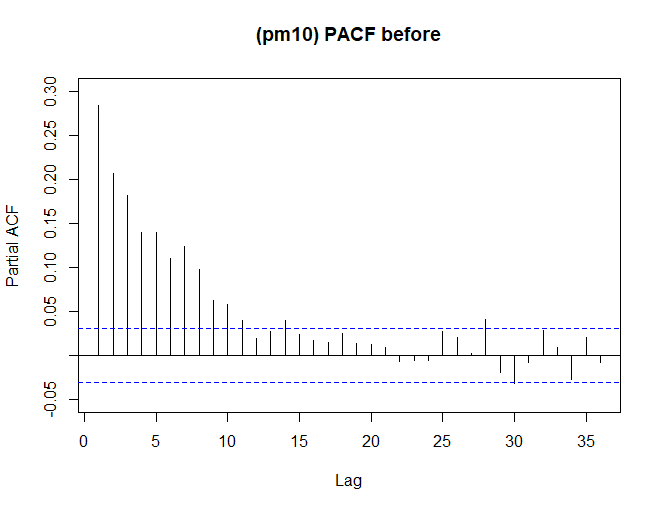 | 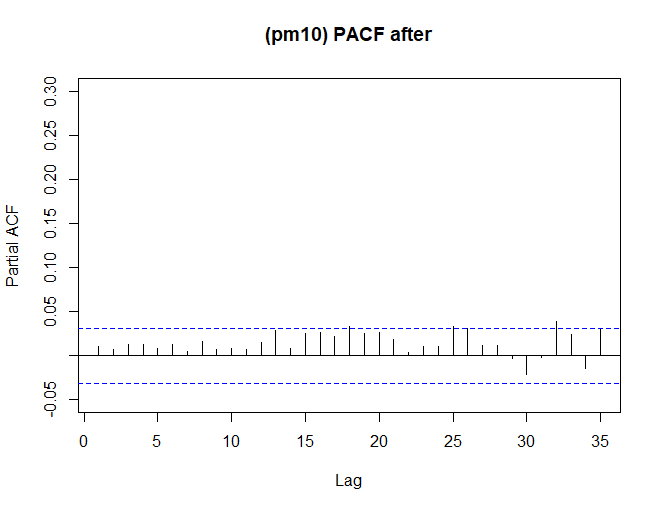 |
| [C] AIC: 17504 | [D] AIC: 16415 |
| 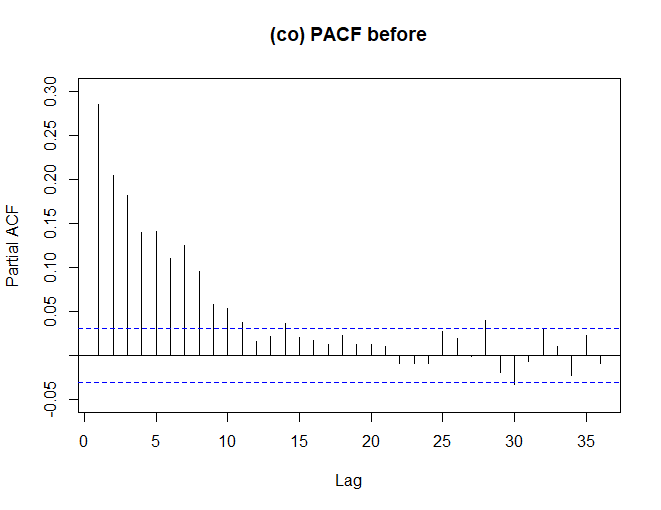 | 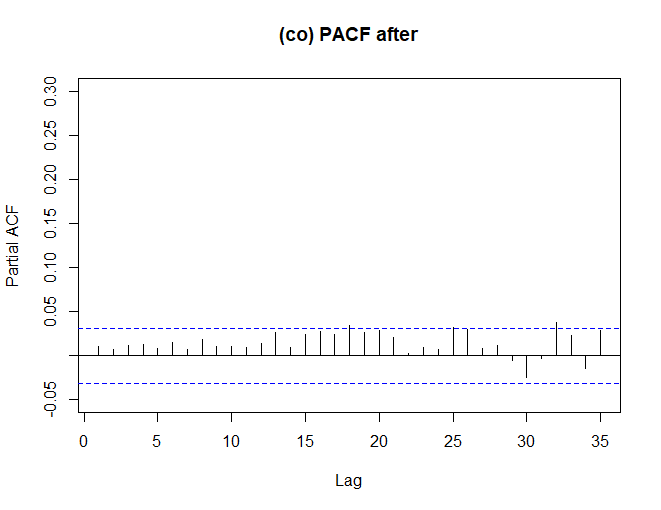 |
| [E] AIC: 17506 | [F] AIC: 16426 |

**Figure 8.** Partial autocorrelation plots (PACF) for deviance residual and Akaike information criterion (AIC) values from final PM_2.5_ (A, B), PM_10_ (C, D) and CO (E, F) models before (A, C, E) and after (B, D, F) adjusting for autocorrelation. The vertical lines represent the extent of correlation (y-axis) between reported RSV infections on the present day and the specified lag (in days) on the x-axis. The area bounded by the horizontal blue dashed lines represents the 95% confidence bands. Autocorrelation is present when any of the vertical lines lie outside the area denoted by the blue dashed lines. The presence of autocorrelation, if not adequately addressed, could lead to potential confounding that results in inaccurate effect estimates.
